# Exploring the relationship between melanopsin gene variants, sleep, and markers of brain health

**DOI:** 10.1002/dad2.70056

**Published:** 2025-01-16

**Authors:** Ayeisha Milligan Armstrong, Eleanor O'Brien, Tenielle Porter, Vincent Dore, Pierrick Bourgeat, Paul Maruff, Christopher C. Rowe, Victor. L. Villemagne, Stephanie R. Rainey‐Smith, Simon M. Laws, the AIBL Research Group

**Affiliations:** ^1^ Centre for Precision Health Edith Cowan University Joondalup Western Australia Australia; ^2^ Collaborative Genomics and Translation Group, School of Medical and Health Sciences Edith Cowan University Joondalup Western Australia Australia; ^3^ Curtin Medical School Curtin University, Kent St. Bentley Western Australia Australia; ^4^ Australian E‐Health Research Centre CSIRO Herston Queensland Australia; ^5^ Department of Molecular Imaging and Therapy and Centre for PET Austin Health Heidelberg Victoria Australia; ^6^ Australian E‐Health Research Centre CSIRO Parkville Victoria Australia; ^7^ Florey Institute of Neuroscience and Mental Health The University of Melbourne Parkville Victoria Australia; ^8^ Cogstate Ltd. Melbourne Victoria Australia; ^9^ Department of Psychiatry University of Pittsburgh Pittsburgh Pennsylvania USA; ^10^ Centre for Healthy Ageing, Health Futures Institute Murdoch University Murdoch Western Australia Australia; ^11^ Alzheimer's Research Australia Sarich Neuroscience Research Institute Nedlands Western Australia Australia; ^12^ School of Psychological Science University of Western Australia Crawley Western Australia Australia; ^13^ School of Medical and Health Sciences Edith Cowan University Joondalup Western Australia Australia

**Keywords:** amyloid, cognition, melanopsin, MRI, sleep

## Abstract

**INTRODUCTION:**

Melanopsin is a photopigment with roles in mediating sleep and circadian‐related processes, which are often disrupted in Alzheimer's disease (AD). Melanopsin also impacts cognition and synaptogenesis. This study investigated the associations between melanopsin genetic variants, sleep, and markers of brain health.

**METHODS:**

Linear regression analyses examined the relationship of single‐nucleotide polymorphisms (SNPs) within the melanopsin gene (*OPN4*), with cortical amyloid beta (Aβ), cognition, brain volumes, and self‐reported sleep traits in cognitively unimpaired older adults. Further analyses assessed whether sleep traits x *OPN4* SNP interactions were associated with markers of brain health.

**RESULTS:**

*OPN4* SNPs rs2355009 and rs3740334 were associated with attention and processing speed and ventricular volume and language, respectively. Furthermore, rs3740334 and rs1079610 showed significant interactions with sleep traits in association with language.

**DISCUSSION:**

This study shows associations of *OPN4* genetic variants with markers of brain health, and suggests that these variants interact with sleep to exacerbate cognitive effects.

**Highlights:**

- The relationships between melanopsin gene (*OPN4*) variants and markers of brain health were examined cross‐sectionally in cognitively unimpaired older individuals.
- Variation within *OPN4*is associated with differences in cognition and ventricular volume.
- rs2355009 and rs3740334 show small–moderate associations with differences in attention and processing speed. Further to this, rs2355009 and rs3740334 were associated with ventricular volumes and language performance, respectively.
- The interactions between rs3740334 and rs1079610 and sleep traits also showed small–moderate associations with differences in language performance.

## 1 BACKGROUND

Melanopsin is a photopigment expressed on the cellular membranes of intrinsically photosensitive retinal ganglion cells (ipRGCs) and is maximally sensitive to light at ≈480 nm (blue light).^1^, ^2^ These melanopsin‐expressing ipRGCs mediate non–image‐forming visual functions, including entraining of circadian rhythm, pupillary light responses, endocrine regulation, and sleep–wake cycles.^3^ The ipRGCs project directly into sub‐cortical brain regions, including the suprachiasmatic nucleus (SCN),^4^ olivary pretectal nucleus,^5^ perihabenular nucleus,^6^ supraoptic nucleus,^7^ and intergeniculate leaflet.^8^ Although ipRGC loss occurs with age, it is more pronounced in Alzheimer's disease (AD), with ≈25% fewer cells than in age‐matched healthy controls.^9^ In addition to amyloid beta (Aβ) deposition, post‐mortem AD retinas also show GC degeneration and thinning of the retinal nerve fiber layer, suggesting ipRGC vulnerability to AD pathologies, potentially from Aβ toxicity or retrograde degeneration from the SCN.^10^, ^11^

Sleep disruptions, including altered sleep architecture, sleep–wake cycles, and circadian rhythms, are common in AD.^12^, ^13^ Poor sleep is associated with increased brain Aβ burden and poorer cognition in older adults, a potentially bidirectional relationship.^14^ The melanopsin‐expressing ipRGCs, which innervate the SCN, are particularly vulnerable to Aβ.^10^ Thus changes in ipRGC function may contribute to sleep and circadian disturbances in AD,^15^ as observed in other neurodegenerative diseases, including Parkinson's disease (PD)^16^ and Huntington's disease (HD).^17^ Therefore, understanding variability in these cells and the influence on AD symptoms or biology may help inform clinical pathological models that link sleep disruption to AD.

Differentiating between the direct and indirect effects of light on cognition in human studies is difficult, as light exerts a wide range of effects on physiological systems, such as sleep and circadian rhythm that can themselves influence cognition.^18^ In mouse models where the melanopsin gene (*OPN4*) has been ablated (*OPN4*–*‐/*–), associations of light exposure with cognition, hippocampal long‐term potentiation, and learning seen in wild‐type mice are no longer observed,^19^, ^20^ suggesting that melanopsin can mediate the effect of light. In *OPN4*–*/*– mice, early cortical synaptogenesis was disrupted with reduced post‐synaptic current frequency and spinal density of pyramidal neurons.^7^ These *OPN4*–*/*– mice also showed reduced rates of learning as adults, demonstrating a role for melanopsin in the promotion of synaptogenesis by light sensation in early life. In humans, prior exposure to different wavelengths of light in an experimental setting altered executive brain responses of the pre‐frontal cortex measured by functional magnetic resonance imaging (fMRI) during the testing phase. These cognitive effects were attributed to melanopsin, as the study was designed to allow for cones and rods to reset between adaptation and testing (70 min in the dark).^21^ In healthy adults, exposure to blue light can improve acute working memory.^19^, ^20^, ^22^ Taken together, these studies suggest a role for melanopsin in mediating cognition.

**RESEARCH IN CONTEXT**

1. **Systematic review**: The authors reviewed the literature from relevant databases (e.g., PubMed). Animal studies show that abolishment of melanopsin results in altered cognitive processes and synaptogenesis. Melanopsin moderates physiological systems that are often disrupted in Alzheimer's disease, including sleep homeostasis and circadian rhythms. However, the effects of melanopsin genetic variation on markers of brain health and the interaction between sleep traits and melanopsin genetic variation on markers of brain health have not been examined.
2. **Interpretation**: The findings provide novel evidence for a role of variation within the melanopsin gene, both directly and in interaction with sleep traits on cognition, particularly language.
3. **Future directions**: Validation of these findings in other cohorts and further longitudinal studies investigating the relationship between melanopsin gene (*OPN4*) variants and markers of brain health is needed. Functional genetic analyses elucidating the molecular underpinnings of these associations are also required. Finally, the study highlights the importance of considering gene by environment interactions in interventional studies.

Melanopsin‐expressing ipRGCs have also been implicated in sleep homeostasis. A case study of delayed sleep–wake phase disorder was found to be caused by a rare deleterious mutation in the *OPN4* gene (rs143641898), which in vitro renders melanopsin non‐functional.^23^ People living with age‐related macular degeneration or glaucoma have reduced ipRGC function, as measured by pupil‐response testing, which in turn is associated with disordered sleep measures.^24^, ^25^ These studies are supported by experiments in mouse models, which report that sleep–wake patterns in *OPN4*–/– mice are disrupted.^26^, ^27^ Further to this, it has been suggested previously that because melanopsin‐expressing ipRGCs are altered in AD, PD, and HD and also innervate the SCN, considered the “master circadian pacemaker,” they may be in part responsible for sleep and circadian disturbances observed in these neurodegenerative disorders.^10^, ^16^, ^17^

Considered together, data from these studies suggest a role of melanopsin in mediating the effects of light on cognition and sleep–wake cycles. They also suggest involvement of melanopsin in the disruption of circadian rhythm and sleep–wake cycles observed in AD. The purpose of this study was to investigate the association of *OPN4* gene variants and self‐reported sleep quality measures with cognition, brain volumes, and cortical Aβ levels. Data from the Australian Imaging, Biomarker and Lifestyle (AIBL) Flagship Study of Ageing was used to investigate associations between *OPN4* gene variants, sleep quality, and markers of brain health in cognitively unimpaired (CU) participants 60 years of age or older. Analyses were also conducted to assess whether the interaction between sleep traits and *OPN4* gene variants was associated with markers of brain health.

## 2 METHODS

### 2.1 Study participants

This analysis utilized cross‐sectional data from participants enrolled in the AIBL Study.^28^ The current study included 614 CU participants 60 years of age or older who had self‐reported sleep quality, genetic, brain Aβ burden, and covariate data available. The sample size for subsequent analyses varied depending on the outcome variable being considered: for investigating sleep and Aβ burden, all 614 participants were included, whereas 571 of the 614 participants had cognitive composite scores and 358 had brain volume measures collected at the same timepoint as the sleep quality data (Table 1). The AIBL Study is approved by ethics committees at Austin Health, St Vincent's Health, Hollywood Private Hospital, Murdoch University, and Edith Cowan University. Participants provided written informed consent prior to undergoing study procedures.

**TABLE 1:** Participant demographics.

	Total	Cognition data available	Brain volume data available
	(*N* = 614)	(*N* = 571)	(*N* = 358)
**Age**, mean (SD)	74.3 (5.53)	74.3 (5.56)	74.4 (5.32)
**Sex,** *N* (%)			
Female	339 (55.2%)	317 (55.5%)	209 (58.4%)
Male	275 (44.8%)	254 (44.5%)	149 (41.6%)
**Centiloid**	13.8 (30.2)	13.8 (30.2)	16.4 (31.1)
**Aβ Status**, *N* (%)			
Low	473 (77.0%)	440 (77.1%)	269 (75.1%)
Abnormally High	141 (23.0%)	131 (22.9%)	89 (24.9%)
***APOE* ε4**, N (%)			
Absent	463 (75.4%)	433 (75.8%)	272 (76.0%)
Present (1/2x ε4 alleles)	151 (24.6%)	138 (24.1%)	86 (24%)
**BMI**, mean (SD)	26.4 (4.18)	26.4 (4.16)	26.3 (4.06)
**CVD**, mean (SD)	0.586 (0.684)	0.592 (0.686)	0.559 (0.662)
**GDS**, mean (SD)	1.21 (1.55)	1.20 (1.55)	1.25 (1.63)
***OPN4* SNPS *N* (%)**			
**rs1079610**			
CC	77 (12.5%)	74 (13.0%)	49 (13.7%)
CT	276 (45.0%)	254 (44.5%)	153 (42.7%)
TT	261 (42.5%)	243 (42.6%)	156 (43.6%)
**rs2355009**			
AA	543 (88.4%)	506 (88.6%)	316 (88.3%)
GA	69 (11.2%)	63 (11.0%)	40 (11.2%)
GG	2 (0.3%)	2 (0.4%)	2 (0.6%)
**rs2736689**			
AA	165 (26.9%)	151 (26.4%)	100 (27.9%)
AC	298 (48.5%)	275 (48.2%)	162 (45.3%)
CC	151 (24.6%)	145 (25.4%)	96 (26.8%)
**rs2803554**			
AA	11 (1.8%)	10 (1.8%)	4 (1.1%)
AC	155 (25.2%)	142 (24.9%)	94 (26.3%)
CC	448 (73.0%)	419 (73.4%)	260 (72.6%)
**rs3740334**			
CC	18 (2.9%)	18 (3.2%)	10 (2.8%)
CT	135 (22.0%)	128 (22.4%)	83 (23.2%)
TT	461 (75.1%)	425 (74.4%)	265 (74.0%)
**rs11202106**			
AA	78 (12.7%)	74 (13.0%)	46 (12.8%)
AG	263 (42.8%)	239 (41.9%)	150 (41.9%)
GG	273 (44.5%)	258 (45.2%)	162 (45.3%)
**Sleep quality traits, mean (SD)**			
**Latency** (min)	21.7 (24.5)	21.9 (25.0)	22.7 (27.1)
**Duration** (h)	6.92 (1.16)	6.90 (1.16)	6.92 (1.20)
**Efficiency**, %	80.4 (13.2)	80.2 (13.3)	80.5 (13.8)
**Disturbances**	8.90 (4.01)	8.81 (4.02)	8.99 (3.95)
**Daytime dysfunction**	0.995 (1.06)	1.01 (1.07)	0.969 (1.03)
**Global PSQI**	6.34 (3.38)	6.39 (3.41)	6.35 (3.29)

*Notes*: Baseline demographics of AIBL participants included in the current study. Of the 614 participants with complete genetic, self‐reported sleep measures, covariate data, and brain Aβ burden data available, 571 had cognition data and 358 also had brain volume data. All values are represented as mean (SD) unless otherwise stated. Aβ Status classified at a threshold of abnormally high >20 Centiloids.

Abbreviations: Aβ, amyloid beta; AIBL, Australian Imaging, Biomarker and Lifestyle Flagship Study of Ageing; *APOE* ε4, apolipoprotein E ε4 allele; BMI, body mass index; CVD, cardiovascular disease (score); GDS, Geriatric Depression Scale (score); h, hours; min, minutes; *OPN4*, melanopsin gene; PSQI, Pittsburgh Sleep Quality Index; SD, standard deviation; SNP, single‐nucleotide polymorphism.

### 2.2 Sleep quality trait measures

Self‐reported sleep quality was assessed using the Pittsburgh Sleep Quality Index (PSQI), a 19‐item questionnaire, from which seven sleep quality measures were calculated. These can be summed to derive a global PSQI score.^29^ Increasing values of the global PSQI score indicate declining sleep quality, with a score greater than 5 typically representing poor sleep.^29^ For the current study, the following measures were analyzed: sleep‐onset latency (minutes to fall asleep), sleep duration (hours), sleep disturbances (quantity), sleep efficiency (time in bed spent asleep expressed as a percentage), daytime dysfunction (quantity), and global PSQI. All sleep quality measures were analyzed as continuous variables. Sleep duration was additionally investigated as a categorical variable (short: <6 h, optimal: 6–8 h, and long: >8 h) based on the previously reported inverted U‐shaped relationship between sleep duration and cognition (i.e., lower cognition with abnormally short or long sleep).^30^

### 2.3 Brain imaging

Cortical Aβ levels were quantified using positron emission tomography (PET) imaging with one of five Aβ tracers: ^11^C‐Pittsburgh compound B, ^18^F‐flutemetamol, ^18^F‐florbetapir, ^18^F‐Florbetaben, or ^18^F‐NAV4694. Images were analyzed using CapAIBL^31^ to generate tracer‐specific standardized uptake value ratios (SUVRs), which were then transformed into Centiloids (CLs).^32^ Participants’ Aβ status was classified as either low or abnormally high (>20 CL).

Magnetic resonance imaging (MRI) scans were used to estimate brain volumes at T1 using the magnetization‐prepared rapid gradient echo (MPRAGE) protocol. MRI scans were acquired using six scanners across four sites. This was followed by estimating cortical brain volumes (gray matter, white matter, and ventricular and hippocampal volumes) using CurAIBL.^33^, ^34^ Brain volumes were corrected for both intracranial volume and MRI scanner.

### 2.4 Cognition

Participants underwent comprehensive neuropsychological assessment using a cognitive test battery.^28^, ^35^, ^36^ The current study assessed six cognitive composite scores, reflecting domains known to be abnormal in the early stages of AD: episodic recall, recognition memory, executive function, language, attention and processing speed,^36^ and the AIBL Preclinical Alzheimer's Cognitive Composite (PACC).^35^

### 2.5 Genotyping and SNP selection

DNA was extracted from whole blood using QIAamp DNA blood spin‐column kits (Qiagen, Valencia, CA, USA) with apolipoprotein E (*APOE*) genotypes determined by Taq Man genotyping assays (Life Technologies, USA).^28^ Genome‐wide single‐nucleotide polymorphism (SNP) genotype data were obtained from Axiom Precision Medicine Diversity Arrays (Applied Biosystems) with subsequent imputation using the TOPMed Imputation Server.^37^ PLINK^38^ was used to filter out samples and SNPs with >2% missing genotypes, minor allele frequency (MAF) <0.05, and deviation from Hardy‐Weinberg equilibrium (*p* < 1 × 10^−6^). SNPs within and 10 kbp upstream of the *OPN4* gene (GRCh38 chr10:86653495‐86666460) were extracted from the imputed data set and linkage disequilibrium pruned (*r* ^2^ cutoff of 0.8, window of 20 kb) using PLINK.^38^ Six SNPs, providing full coverage of *OPN4*, were taken forward for analysis (Table 2; see Table S1 for linkage disequilibrium scores [in AIBL] between SNPs).

**TABLE 2:** OPN4 SNPs.

rsID	Position (GRCh38)	Major allele	Minor allele	Gene consequences
rs1079610	chr10:86662359	T	C	Missense variant
rs2355009	chr10:86655261	A	G	Intron variant
rs2736689	chr10:86653675	C	A	2‐kb upstream variant
rs2803554	chr10:86665798	C	A	3′ untranslated region variant
rs3740334	chr10:86659255	T	C	Intron variant
rs11202106	chr10:86654813	G	A	Synonymous variant

*Notes*: *OPN4* SNPs investigated in the current study, major and minor alleles.

Abbreviations: GRCh38, Genome Reference Consortium Human Build 38; kb, kilobase; *OPN4*, melanopsin gene; SNP, single‐nucleotide polymorphism.

### 2.6 Statistical analysis

Statistical analyses were undertaken using R (4.3.2), run in RStudio (2023.09.1+494) for MacOS. Demographic and clinical characteristics were estimated for the complete sample, and for the subsample for which cognitive composite scores and MRI brain volumes were available.

Linear regression analyses were undertaken using the *lm* function to investigate the relationship between *OPN4* SNPs and two sets of outcome variables: (1) markers of brain health (brain Aβ, cognition, and brain volume), and (2) sleep traits (sleep‐onset latency, sleep duration, sleep disturbances, sleep efficiency, daytime dysfunction, and global PSQI). Separate models were run for each combination of SNP and outcome variable. For all analyses, age and sex were included as covariates, and the dominant genetic model (carriage of minor allele vs major allele homozygotes) was assumed. In addition, when markers of brain health were analyzed, *APOE*‐ε4 status (binary; absent/present) was included as a covariate. Brain Aβ status (low/abnormally high) was included as a covariate in analyses where sleep traits, cognition, or brain volumes were the outcome variables. Cardiovascular disease (CVD) history (summed score of history of heart disease, hypertension, angina, and stroke), body mass index (BMI), and Geriatric Depression Scale (GDS) scores were included as covariates in all analyses of sleep traits.^14^ The models are defined below:

$$\begin{array}{c} Brain\;A\beta \;burden∼OPN4\;SNP+Age+Sex+APOE-\varepsilon 4\\ Cognition/Brain\;Volume∼OPN4\;SNP+Age+Sex+APOE-\varepsilon 4\\ \;\;+\;A\beta status\\ Sleep\;Trait\;Measure∼OPN4\;SNP+Age+Sex+A\beta status\\ \;\;+\;CVD+GDS+BMI \end{array}$$

To investigate whether sleep traits moderate the relationship between *OPN4* SNPs and markers of brain health (as the outcome variable), linear regression models were run with a SNP x sleep trait interaction term. These models included age, sex, *APOE* ε4 status, CVD, BMI, and GDS as covariates. Brain Aβ status was additionally included as a covariate in analyses where cognition or brain volumes were the outcome variables. The models are defined below:

$$\begin{array}{c} BrainA\beta burden∼OPN4SNPxSleep\;Trait\;Measure+Age+Sex\\ \;\;+\;APOE-\varepsilon 4+CVD+BMI+GDS\\ Cognition/Brain\;Volume∼OPN4\;SNPx\;Sleep\;Trait\;Measure+Age+Sex\\ \;\;+\;APOE-\varepsilon 4+A\beta status+CVD+BMI+GDS \end{array}$$

Correction for multiple testing was performed using the false discovery rate (FDR) method,^39^ where *p*‐values for terms of interest (*OPN4* SNP or *OPN4* SNP x sleep) were adjusted within each outcome variable for each set of models (*q*‐values). Effect sizes were calculated as Cohen's *d*.

## 3 RESULTS

### 3.1 Demographics

Group demographic characteristics, genotype frequencies, and summary sleep characteristics are summarized in Table 1, for the entire study cohort (i.e., individuals for which sleep quality, genetics, and brain Αβ levels were available,  *n *= 614), and for the subgroups that also had available cognitive composite scores (*n* = 571) and estimates of brain volume (*n* = 358).

### 3.2 Relationship of *OPN4* SNP variation with markers of brain health and sleep traits

#### 3.2.1 *OPN4* SNPs and cognition

**FIGURE 1:**
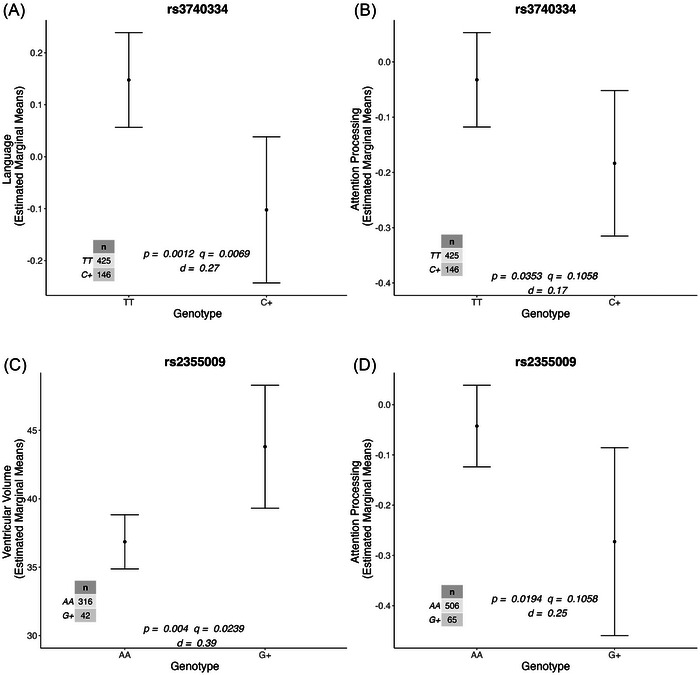
Significant associations between *OPN4* SNPs and cross‐sectional markers of brain health. Representation of the estimated marginal means of (A) rs3740334 on language, (B) rs3740334 on attention and processing speed, (C) rs2355009 on ventricular volume, and (D) rs2355009 on attention and processing speed. All SNPs were run under a dominant genetic model (major allele homozygotes vs carriers of minor allele). Error bars represent the 95% confidence intervals of estimated marginal means. False discovery rate corrected *p*‐values are represented as *q*‐values. Effect sizes are represented as Cohen's *d*. Covariates: age, sex, *APOE* ε4, and Aβ status. Aβ, amyloid beta; *APOE* ε4, apolipoprotein E ε4 allele; *OPN4*, melanopsin gene; SNP, single‐nucleotide polymorphism.

Analyses indicated nominally significant associations with small–moderate effect sizes of rs2355009 and rs3740334 with attention and processing speed (Figure 1; Table 3), although these did not survive FDR correction (rs2355009 [*β = −*0.230, standard error (*SE)* *=* 0.098, *p =* 0.019, *q =* 0.106, *d =* 0.25]; rs3740334 [*β = −*0.151, *SE =* 0.072, *p =* 0.035, *q =* 0.106, *d =* 0.17]). The rs3740334 SNP was significantly associated with language following FDR correction (*β = −*0.250, *SE =* 0.077, *p =* 0.001, *q =* 0.007, *d =* 0.27) (Figure 1; Table 3). Carriage of the rs3740334 minor allele (C+) was associated with poorer performance in the language and attention and processing speed domains. Carriers of the rs2355009 minor allele (G+) had lower scores for attention and processing speed. No other significant associations were observed for the remaining SNPs and cognitive domains (Table S2).

**TABLE 3:** *OPN4* SNPs nominally associated with markers of brain health.

Brain health trait	SNP	*β*	SE	*p*‐value	*q*‐value
Attention and processing speed	rs2355009	−0.230	0.098	0.019	0.106
	rs3740334	−0.151	0.072	0.035	0.106
Language	rs3740334	−0.250	0.077	0.001	**0.007**
Ventricular volume	rs2355009	6.950	2.398	0.004	**0.024**

*Notes*: Cross‐sectional linear regression results for nominally significant associations between *OPN4* SNPs and brain health traits. All SNPs were run under a dominant genetic model (major allele homozygotes vs minor allele carriers). The *β* coefficient represents the effect of carriage of the minor allele on the stated outcome variable, and SE represents the standard error of this estimate. The *q*‐value represents the false discovery rate corrected *p*‐value of the stated estimate, with associations that remained significant following this correction bolded. Covariates: age, sex, *APOE ‐*ε4, and Aβ status.

Abbreviations: Aβ, amyloid beta; AD, Alzheimer's disease; *APOE* ε4, apolipoprotein E ε4 allele*; OPN4*, melanopsin gene; SE, standard error; SNP, single‐nucleotide polymorphism.

#### 3.2.2 *OPN4* SNPs and brain imaging measures

There were no significant associations observed between *OPN4* SNPs and brain Aβ burden. The rs2355009 SNP was significantly associated with ventricular volume after FDR correction (*β = *6.950, *SE = *2.398, *p =* 0.004, *q =* 0.024, *d =* 0.39) (Figure 1; Table 3). Consistent with the result for attention and processing speed above, the minor allele of rs2355009 conferred risk, with the G+ genotype group showing larger ventricular volumes (Figure 1). No other significant associations were observed between SNPs and brain volume measures (Table S2).

#### 3.2.3 *OPN4 SNPs* and sleep traits

No significant associations were observed between *OPN4* SNPs and sleep measures (Table S3).

### 3.3 The interaction of *OPN4* SNPs and sleep measures on markers of brain health

**FIGURE 2:**
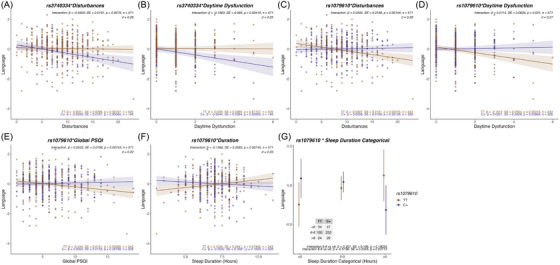
Interaction of *OPN4* SNPs and sleep measures on language. Representation of linear regression analyses that showed FDR significant interactions between different genotype groups of the rs3740334 SNP and (A) sleep disturbances and (B) daytime dysfunction, with effects on cross‐sectional measures of the language cognitive domain. In addition, representation of linear regression analyses showed FDR significant interactions between different genotype groups of the rs1079610 SNP and (C) sleep disturbances, (D) daytime dysfunction, (E) global PSQI, and (F) sleep duration as a continuous and (G) categorical variable with effects on cross‐sectional measures of the language cognitive domain. The main effect of the interaction term SNP*Sleep trait measure is denoted as well as the group effect estimated marginal means for the different genotype groups. For sleep duration as a categorical analysis the difference between optimal and short and optimal and long sleep is denoted. Effect sizes are represented as Cohen's *d*. Covariates: age, sex, *APOE* ε4, Aβ status, CVD, BMI, and GDS. Aβ, amyloid beta; *APOE* ε4, apolipoprotein E ε4 allele; BMI, body mass index; CVD, cardiovascular disease (score); GDS, Geriatric Depression Scale (score); *OPN4*, melanopsin gene; PACC, Preclinical Alzheimer's Cognitive Composite; PSQI, Pittsburgh Sleep Quality Index; SNP, single‐nucleotide polymorphism.

There were 32 models for which the SNP * Sleep measure interaction term was nominally significantly associated with markers of brain health (Table 4; full results in Table S4). Of these, six associations remained significant following FDR correction, all for the cognitive domain of language (Figure 2; Table 4).

**TABLE 4:** Nominally significant OPN4*Sleep trait interactions.

Brain health trait	SNP	Sleep trait	*β*	SE	*p*‐value	*q*‐value
AIBL PACC	rs1079610	Duration	−0.111	0.046	0.016	0.273
	rs1079610	Efficiency	−0.009	0.004	0.023	0.273
	rs1079610	Global PSQI	0.037	0.016	0.019	0.273
	rs2736689	Disturbances	−0.031	0.015	0.034	0.306
Attention and processing speed	rs2355009	Latency	0.010	0.004	0.019	0.481
	rs3740334	Disturbances	−0.040	0.018	0.027	0.481
Episodic recall	rs1079610	Disturbances	0.039	0.016	0.015	0.186
	rs1079610	Duration	−0.122	0.056	0.029	0.258
	rs1079610	Efficiency	−0.013	0.005	0.010	0.180
	rs1079610	Global PSQI	0.054	0.019	0.004	0.159
	rs2736689	Disturbances	−0.038	0.018	0.038	0.271
Executive function	rs11202106	Disturbances	−0.040	0.016	0.012	0.148
	rs11202106	Global PSQI	−0.041	0.019	0.030	0.274
	rs11202106	Latency	−0.007	0.003	0.012	0.148
	rs2803554	Latency	0.008	0.003	0.003	0.122
Language	rs1079610	Daytime dysfunction	0.211	0.064	0.001	**0.019**
	rs1079610	Disturbances	0.056	0.017	0.001	**0.019**
	rs1079610	Duration	−0.157	0.058	0.007	**0.047**
	rs1079610	Efficiency	−0.010	0.005	0.046	0.167
	rs1079610	Global PSQI	0.053	0.020	0.007	**0.047**
	rs2355009	Duration	0.171	0.082	0.038	0.153
	rs2736689	Disturbances	−0.041	0.019	0.031	0.138
	rs2803554	Efficiency	0.013	0.006	0.028	0.138
	rs3740334	Daytime dysfunction	−0.198	0.069	0.004	**0.047**
	rs3740334	Disturbances	−0.051	0.019	0.008	**0.047**
Recognition	rs1079610	Duration	−0.127	0.052	0.015	0.314
	rs2736689	Duration	0.138	0.058	0.017	0.314
Gray matter volume	rs2355009	Disturbances	1.495	0.684	0.029	0.990
Hippocampus volume	rs1079610	Daytime dysfunction	−0.124	0.053	0.020	0.703
Ventricular volume	rs11202106	Duration	2.570	1.296	0.048	0.608
	rs2355009	Daytime dysfunction	−5.794	2.874	0.045	0.608
White matter volume	rs2803554	Daytime dysfunction	−7.069	2.423	0.004	0.135

*Notes*: Cross‐sectional linear regression results for nominally significant associations between brain health traits and the *OPN4* SNP*Sleep Trait interaction term. All SNPs were run as a dominant genetic model (major allele homozygotes vs minor allele carriers). *β* represents the coefficient of the interaction between the SNP and sleep trait measure, and SE represents the standard error of this estimate. The *q*‐value represents the false discovery rate correction of the *p*‐value for each of these interaction terms on listed outcome variables, with those that remain significant following correction bolded. Covariates: age, sex, *APOE* ε4, Aβ status, CVD, BMI, and GDS.

Abbreviations: Aβ, amyloid beta; AD, Alzheimer's disease; *APOE* ε4, Apolipoprotein E ε4 allele; BMI, body mass index; CVD, cardiovascular disease (score); GDS, Geriatric Depression Scale (score); *OPN4*, melanopsin gene; PACC, Preclinical Alzheimer's Cognitive Composite; PSQI, Pittsburgh Sleep Quality Index; SE, standard error; SNP, single‐nucleotide polymorphism.

Both rs3740334 and rs1079610 showed small–moderate but significant interactions with sleep measures in association with language. For the rs3740334 SNP, interactions with sleep disturbances (*β = −*0.05, *SE =* 0.019, *p =* 0.008, *q = *0.047, *d =* 0.26) and daytime dysfunction (*β = −*0.198, *SE = *0.069, *p = *0.004, *q = *0.047, *d =* 0.25) were significantly associated with the language domain (Table 4). Among carriers of the minor allele (C+), language scores decreased with poorer sleep quality (increased sleep disturbances and daytime dysfunction), whereas there was no significant association of these sleep measures with language in the homozygotes of the major allele (TT) (Figure 2). The interactions of the rs1079610 SNP with sleep disturbance *(β = *0.056, *SE = * 0.017, *p = *0.001, *q = *0.019, *d =* 0.28), daytime dysfunction (*β =* 0.211, *SE = *0.064, *p = *0.001, *q = *0.019, *d =* 0.27), global PSQI (*β =* 0.053, *SE = *0.020, *p = *0.007, *q = *0.047, *d = *0.22), and sleep duration (*β = −*0.157, *SE = *0.058, *p = *0.007, *q = *0.047, *d =* 0.23) were also significantly associated with language (Table 4). TT homozygotes had decreasing language scores with worsening sleep measures for sleep disturbance, daytime dysfunction, and global PSQI, whereas these association were not significant for people with the C+ genotype (Figure 2). For sleep duration, the relationship was more complex, with TT individuals showing poorer language scores than C+ individuals when sleep duration was shorter, and better language skills than C+ individuals when sleep duration was longer (Figure 2). This, combined with the previously observed inverted U‐shaped relationship of sleep duration with cognition, prompted further investigation of the association by re‐running the interaction model with sleep duration as a categorical variable: short (<6 h), optimal (6–8 h), or long (>8 h). This analysis showed that the TT homozygotes had significantly better language scores than C+ individuals, with sleep duration of greater than 8 h, an association not observed in individuals with short or optimal sleep duration.

## 4 DISCUSSION

This study investigated the association of variation within the melanopsin gene *OPN4* with self‐reported sleep quality, and with AD‐related biological, neuronal, and symptomatic characteristics. In addition, the study investigated whether the interactions between *OPN4* SNPs and sleep quality were associated with markers of brain health. Direct associations, with small–moderate effect sizes of two *OPN4* SNPs (rs3740334 and rs2355009) with AD‐related traits, and significant interactions of two *OPN4* SNPs (rs3740334 and rs1079610) with sleep quality traits in association with cognition (specifically language) were observed.

The rs3740334 (T > C) intronic variant^40^ showed direct and indirect associations of small–moderate effect sizes with language and attention and processing speed. ForgeDB (score = 8) and RegulomeDB (score = 1f) scoring matrices predict rs3740334 to have regulatory potential.^41^, ^42^ The rs3740334 SNP has been identified previously as an expression quantitative trait locus (eQTL) for *OPN4* across multiple tissues, with carriage of the C allele associated with reduced expression of *OPN4* in several brain regions.^43^ In the current study, C+ participants had poorer language and attention, and processing cognitive scores overall, as well as poorer language performance with worsening sleep. These results are consistent with previous studies reporting a positive relationship between *OPN4* expression and cognitive performance. Studies have shown that mice completely lacking *OPN4* show reduced learning abilities later in life.^7^ However, given that ours is the first study to report an association between *OPN4* genetic variation and differences in cognitive measures, these results should be validated in other well‐characterized cohorts. Furthermore, in vitro and pre‐clinical in vivo experiments to investigate the mechanism underlying this relationship may provide further explanation for the current observation.

The intronic rs2355009 (A > G) variant is estimated to have high regulatory potential based on ForgeDB (8) and RegulomeDB (1f) scoring matrices. In the current study, the minor allele (G+) was associated with increased brain ventricular volumes and decreased attention and processing speed, both with small–moderate effect sizes. To the best of our knowledge, there are no previous reports of associations between melanopsin and brain volume measures; therefore replication of these findings in other well‐characterized cohorts is required. The potential mechanism through which this association may be explained remains unknown.

The missense variant rs1079610 (T > C) results in a substitution of isoleucine to threonine at position 394.^40^ Previous studies have shown rs1079610 to be associated with altered sleep–wake cycles (by ≈1 h) and pupil response to illuminance.^44^, ^45^ Individuals with the C+ genotype showed increased pupil restriction under conditions of high illuminance as compared to the TT genotype,^44^ suggesting a role for this SNP in moderating melanopsin function, which is known to alter pupillary reflexes. A study in university students found that increased pupil responsiveness was associated with better working memory.^46^ In the current study, the TT genotype interacted with multiple sleep traits, albeit with small–moderate effect sizes, where with worsening sleep quality, poorer language performance was observed. Mechanistically, it may be hypothesized that this SNP, which alters pupillary response to light (itself associated with working memory), may partially explain the observed effects. Although working memory and language are distinct domains, research suggests a connection between them.^47^ This study indicates that TT genotype individuals might benefit more from improved sleep quality than C+ carriers. However, it should also be noted that given the rs1079610 x sleep quality interactions were not associated with either Aβ burden or brain volume measures, this observed effect on language may be the result of functional changes impacting cognition rather than structural. Further study on how this interaction influences language over time is needed. In addition, within AIBL, the cognitive domain of language is assessed using comprehensive neuropsychological assessments; thus, evaluating how these language differences affect perceived language performance could offer more insights. Although in 2014,Lee et al.,^45^ showed that the rs1079610 variant was associated with differences in sleep–wake timings in a younger population of university students, no associations of any *OPN4* SNPs investigated with self‐reported sleep quality traits were observed in the current study of older adults. Our findings are consistent with those of Higuchi et al. (2013),^44^ who found no differences in PSQI measures between the rs1079610 genotypes. The complete functional consequences of the missense variant rs1079610 on melanopsin are not yet understood.

In the current study, there were no observed associations of any of the investigated *OPN4* SNPs with sleep traits, suggesting that the relationships between melanopsin and cognition, and to a lesser extent ventricular volume, are not driven simply by differing sleep traits. However, two SNPs did significantly interact with sleep traits in their associations with cognition, which may reflect differences in the cognitive consequences of poor sleep associated with variation within the *OPN4* gene. Previous studies suggest that *OPN4* genetic variants are associated with differences in sleep–wake times,^23^, ^45^ possibly due to melanopsin ipRGCs innervating the SCN. However, as this study utilized only the PSQI, which does not specifically assess sleep–wake or circadian rhythms, other sleep traits may be influenced by *OPN4* SNPs, particularly in CU individuals. This aligns with a study of Japanese students in which no associations between rs1079610 and PSQI measures were observed.^44^

Studies elucidating the role of melanopsin in cognition are limited. In animal studies, ablation of *OPN4* results in reduced synaptic formation and learning, suggesting a direct role for light sensitization via melanopsin in proper synaptogenesis formation, and subsequent cognitive processes via an oxytocin neuron circuit.^7^ In post‐mortem AD retinas, a loss of melanopsin expressing ipRGCs is observed, although it is unknown how this relates to disease progression.^10^ In the current study, two SNPs found to be associated with cognition are suggested to have regulatory potential, whereas the third is a missense mutation. Given that one of the SNPs in the current study is an eQTL for reduced *OPN4* expression in multiple brain regions, it would be of interest for further studies to investigate whether this reduction in *OPN4* expression is responsible for the reduced cognitive performance observed. Experimental validation using in vitro and in vivo models of these gene variants may also help to elucidate how changes to melanopsin structure or function can contribute to this.

The findings of this study provide novel evidence suggesting that variation within the *OPN4* gene may interact with sleep quality to influence cognition, particularly the language domain. However, the study has some limitations. Although data for sleep quality (PSQI) measures were taken from the same cross‐sectional time point as the cognitive assessments and MRI and PET scans, these tests were not all performed on the same day. It is important to note, however, that changes in intra‐individual PSQI measures across the AIBL Study have been stable longitudinally (data not shown). Therefore, it is not anticipated that this aspect of experimental design would impact our findings. In addition, PSQI measures are self‐reported, meaning they are subjective and reliant upon participants’ recollections and are therefore susceptible to recall bias. Despite this, the PSQI is widely adopted and has been shown previously to be a cost‐effective, reliable, and valid measure of sleep quality.^48^ In addition, although significant sleep disorders, including obstructive sleep apnea, are an AIBL exclusion criterion, undiagnosed cases cannot be discounted. This is a recognized limitation, as sleep apnea has been associated with brain Aβ burden and cognition.^49^ Finally, the AIBL cohort comprises mostly Caucasian population, and therefore, our findings require validation in other ethnically diverse populations, as there are known differences in the effects of genetic variants across different ancestries.^50^

To the best of our knowledge, this is the first study to investigate the association of variation within *OPN4* with variation in markers of brain health. As such, it is important that these findings are validated in other comparable independent cohorts. The associations between *OPN4* variation and cognition, both directly and in interaction with sleep traits, suggest that melanopsin represents a promising avenue of research for understanding factors affecting cognition. Sleep is considered a modifiable lifestyle factor, and our findings suggest that the cognitive benefits of sleep improvement interventions may vary depending on *OPN4* genotype. Such findings are important for informing personalized treatment and prevention of cognitive decline. Functional characterization of *OPN4* variants is needed to elucidate underlying molecular mechanisms. Studies of the influence of these variants on longitudinal assessments of cognition and brain volumes are also warranted.

## CONFLICT OF INTEREST STATEMENT

A.M.A., E.O., T.P., V.D., P.B., S.R.R.S., and S.M.L. report no disclosures. V.L.V. is and has been a consultant or paid speaker at sponsored conference sessions for Eli Lilly, Life Molecular Imaging, ACE Barcelona, and IXICO. P.M. is a full‐time employee of Cogstate Ltd. C.C.R. has served on scientific advisory boards for Bayer Pharma, Elan Corporation, GE Healthcare, and AstraZeneca; has received speaker honoraria from Bayer Pharma and GE Healthcare; and has received research support from Bayer Pharma, GE Healthcare, Piramal Lifesciences, and Avid Radiopharmaceuticals. Author disclosures are available in the Supporting Information.

## CONSENT STATEMENT

Prior to undergoing any study procedures or assessments, all individuals provided written informed consent with respect to procedures and protocols approved by the institutional ethics committees of Austin Health, St. Vincent's Health, Hollywood Private Hospital (now Ramsay Health Care), Murdoch University, and Edith Cowan University.

## Supporting information



Supporting information

Supporting information

Supporting information

Supporting information

Supporting information
